# Changes in short term river flow regulation and hydropeaking in Nordic rivers

**DOI:** 10.1038/s41598-018-35406-3

**Published:** 2018-11-22

**Authors:** Faisal Bin Ashraf, Ali Torabi Haghighi, Joakim Riml, Knut Alfredsen, Jarkko J. Koskela, Bjørn Kløve, Hannu Marttila

**Affiliations:** 1 Water Resources and Environmental Engineering Research Unit, PO Box 4300, 90014 University of Oulu, Oulu, Finland; 2 Royal Institute of Technology (KTH), Brinellvägen 8, 114 28 Stockholm, Sweden; 3 Norwegian University of Science and Technology (NTNU) Vassbygget, 442, Valgrinda, Trondheim Norway; 4 Finnish Environment Institute (SYKE), Mechelininkatu 34a, 00260 Helsinki, PO Box 140, Helsinki, Finland

## Abstract

Quantifying short-term changes in river flow is important in understanding the environmental impacts of hydropower generation. Energy markets can change rapidly and energy demand fluctuates at sub-daily scales, which may cause corresponding changes in regulated river flow (hydropeaking). Due to increasing use of renewable energy, in future hydropower will play a greater role as a load balancing power source. This may increase current hydropeaking levels in Nordic river systems, creating challenges in maintaining a healthy ecological status. This study examined driving forces for hydropeaking in Nordic rivers using extensive datasets from 150 sites with hourly time step river discharge data. It also investigated the influence of increased wind power production on hydropeaking. The data revealed that hydropeaking is at high levels in the Nordic rivers and have seen an increase over the last decade and especially over the past few years. These results indicate that increased building for renewable energy may increase hydropeaking in Nordic rivers.

## Introduction

Hydropower plays a significant role in Europe’s energy market and is gaining further importance in many developing counties since it is a flexible energy source^1^. The Intergovernmental Panel on Climate Change (IPCC) expects the share of low-carbon energy to increase by over three-fold in the stringent RCP 2.6 mitigation scenarios^2^. Moreover, the European Union (EU) is aiming to increase its use of renewable energy to 20% of total energy consumption by 2020^3^. This increasing production of renewables in energy markets demands more flexible management of energy resources. In conjunction with ongoing climate change and its effects on river flows^4,5^, sudden sub-daily flow variations (hydropeaking) and other kinds of river regulation will have additional influences on reservoir regulation and water release patterns. Sudden changes in power production and consumption will most likely be compensated for by hydropower reservoir operation, which may cause hydropeaking in downstream river systems^6,7^. In recent years, managing water resources for more efficient hydropower production and providing hydropower as a back-up source for wind and solar energy has become an important issue in the Nordic countries^8^. Accumulated hydropeaking over a longer period would ultimately result in overall river regime alteration.

River regime alteration is recognized as a key threat to many riverine species and ecosystem services which rivers provide^9–11^. Hydropower causes direct or indirect impacts on river systems by altering the temporal patterns of water flow^12^ and restructuring natural^13–16^ habitats. Damming and regulation of river systems result in homogenization of long-term river dynamics^9,17–19^, and also hydropeaking^20,21^. An increase in hydropeaking would pose challenges in maintaining current river ecological status and thus the potential effects need to be evaluated in more detail for Nordic rivers.

To ensure suitable natural flow conditions for river biota and a sustainable nexus of water, energy, and climate, more knowledge is needed about natural conditions and changes caused by human interventions and potentially by climate change. The European Water Framework Directive (2000/60/EC) and Blueprint (COM(2012)673) encourage definition of sustainable flow conditions for degraded river systems that account for human needs and also water quantity for biotic and recreation needs. Typically, flow variability is observed over a large range of temporal scales ranging from hours to seasons, and is important for maintaining natural hydraulic complexity, sediment transport, hyporheic exchanges, floodplain connections, and habitat structure and complexity^22,23^. In the rapidly changing energy markets in the Nordic countries (introduction of renewable energy sources such as wind, solar, etc.), the intraday flow patterns are being altered. This alteration originates from changes in demand for load-balancing energy, causing sudden changes in river regimes, i.e., hydropeaking. Hydropeaking especially affects fish behavior^24–28^ and has been linked to juvenile fish mortality^29^ and maladaptive behavior in adult fishes and failure of spawning^29–32^. Some previous studies have quantified the impacts of operational changes and climate change on hydropeaking in regulated rivers^33–36^. However, most of these studies have not investigated hydropeaking in Nordic context. And those who have, are limited to small study areas or have examined the effect of catchment properties, while the relationship with hydropower production capacity and other catchment properties has not been considered^20^. Hydropeaking is generally assumed to occur in rivers with a considerable headwater reservoir. Hence most of the earlier studies focus on rivers with large reservoir storage^34,37^, while investigations on run-of-river (ROR) hydropower plants with small reservoir capacity are generally lacking.

Many studies have quantified long-term river regime change by taking into account data with daily or higher resolution^18,38,39^. Fewer studies quantifying river regime alteration have used subdaily data or examined seasonal variations in hydropeaking^21,36,40^. In order to quantify such seasonal variations, it is important that underlying data have short time steps. Observing discharge alterations in rivers at sub-daily resolution can help reveal flow characteristics that might otherwise be overlooked when analyzing discharge data with daily or monthly resolution. Therefore, in the present work we quantified the levels of hydropeaking using high-resolution data (hourly) covering multiple years and large spatial areas for Nordic rivers (Finland, Sweden, Norway). High-frequency variations at a given time were analyzed and seasonal changes in hydropeaking were investigated. We used a statistical analysis approach to quantify and compare hydropeaking measured with respect to hydropower plant type and catchment properties across multiple rivers. In Nordic rivers, there is a conflict between environmental and economic objectives. These countries want to increase the transition towards renewable energy with increased use of hydropower as both a primary and balancing energy source, but at the same time they want to minimize the incidence of hydropeaking due to negative ecological impacts. The aims in this study were to: (i) Determine the current situation as regards hydropeaking in Nordic rivers; (ii) analyze drivers behind the observed changes in river regimes, and (iii) identify the overall driving forces for hydropeaking. In the analyses, the importance of using data with high temporal resolution to analyze flow conditions in regulated river systems was taken into account^21,36^.

## Results

### Overall status of hydropeaking in the region

**Figure 1:**
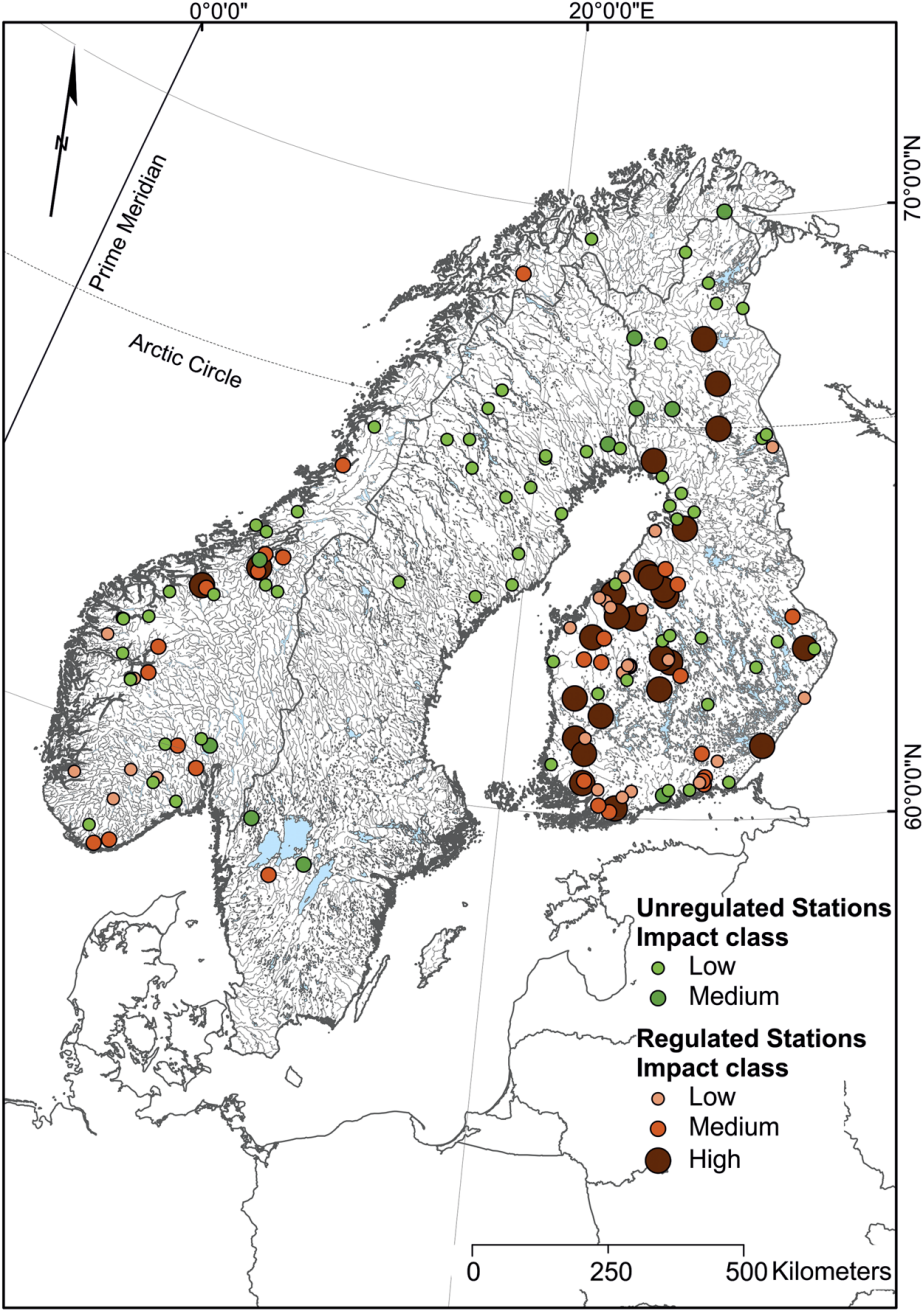
Map of the studied area showing measure and location of hydropeaking in studied sites.

**Figure 2:**
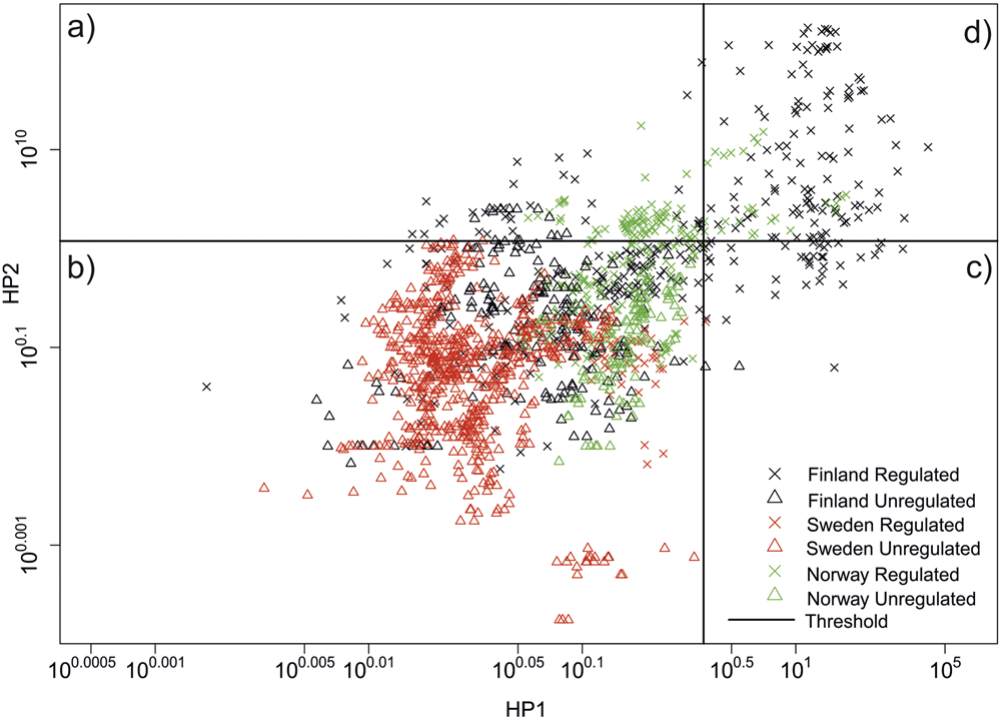
Overall distribution of whole datasets in classes of different hydropeaking impact. Four parts of the graph identified as three different hydropeaking impact class are: (**b**) low impact, (**a**,**c**) medium impact, and (**d**) high impact. Thresholds have been calculated using data from unregulated stations.

The hydropeaking indicator (HP1) ranged between 0 and 2 and the ramping rate indicator (HP2) ranged between 0 and 160 in the whole region. Finnish rivers showed the highest hydropeaking impact, followed by Norwegian and Swedish rivers (Fig. 1). The threshold value in Finland, Sweden, and Norway was found to be 0.29, 0.14 and 0.66, respectively, for the HP1 indicator, and 1.21, 1.01, and 1.34 for the HP2 indicator. In all, 64% of Finnish and 77% of Norwegian regulated rivers exhibited moderate to high levels of hydropeaking. The analysis included only one regulated river gauging station in Sweden with high-resolution (hourly) flow data, which showed low levels of hydropeaking (Figs 1 and 2).

**Figure 3:**
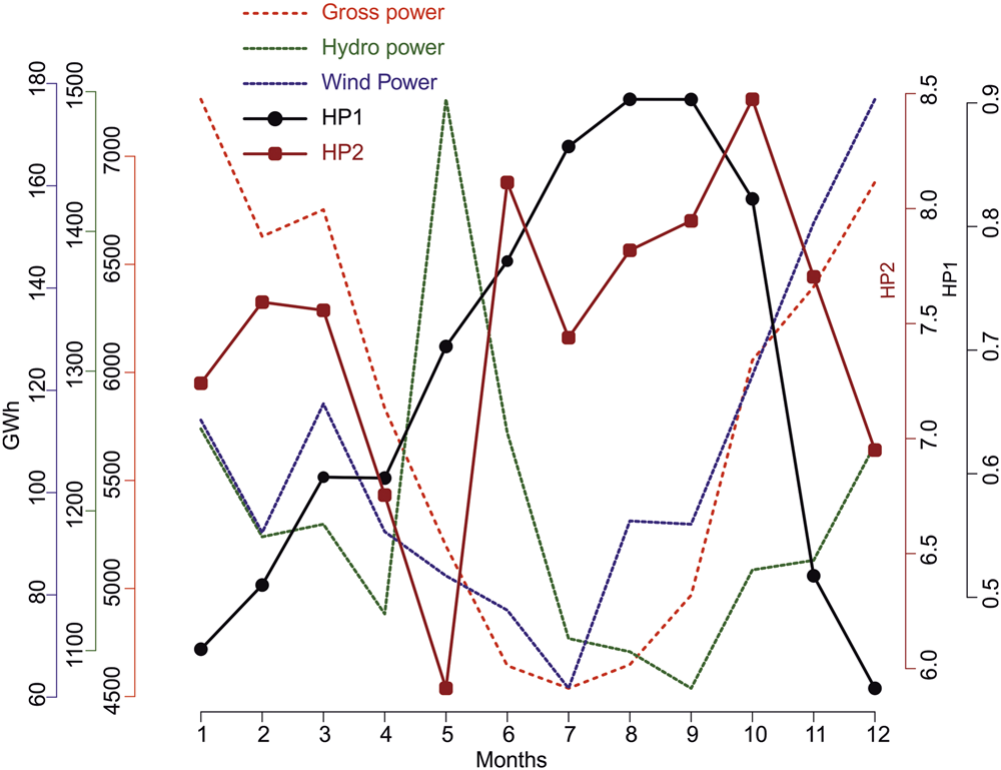
Monthly aggregated power production by different sources and corresponding HP1 and HP2 values in Finland since 2007. Months on X-axis are in the order of January (1) to December (12). Source Finland energy statistics report 2018.

The natural river regime in seasonally snow-covered regions is low flow during snow accumulation and high flow during snowmelt. Hydropeaking varied widely between different seasons in the rivers analyzed in this study. An analysis of aggregated monthly power production (for Finland only) between 2007 and 2016 and its relationship with aggregated hydropeaking values revealed that HP1 and HP2 had higher values in months with low power production and lower values in months with high power production (Fig. 3).

**Figure 4:**
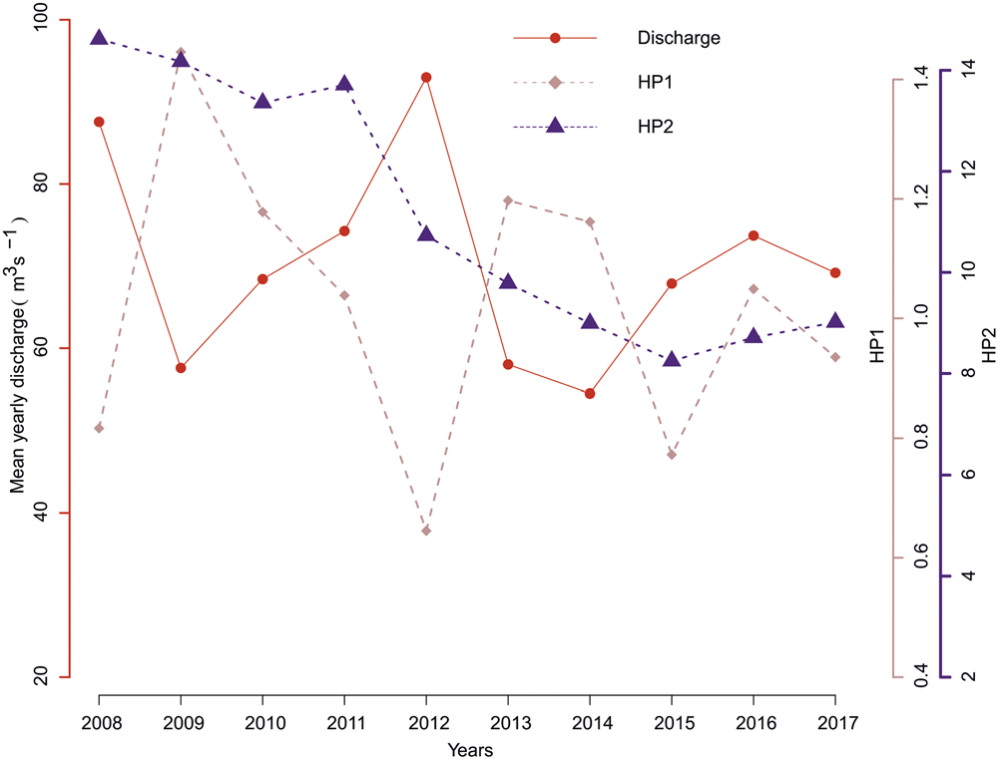
Aggregated yearly mean discharge from the regulated sites on Finnish rivers and corresponding HP1 and HP2 values.

The HP1 values were inversely proportional to aggregated mean discharge from all regulated rivers studied in Finland, whereas HP2 showed no such pattern (Fig. 4). The highest hydropeaking values in Finnish rivers were observed in summer low-flow periods. HP1 and HP2 showed contrasting patterns during snowmelt months, with HP1 rising steadily during the snowmelt months and HP2 dipping sharply.

**Figure 5:**
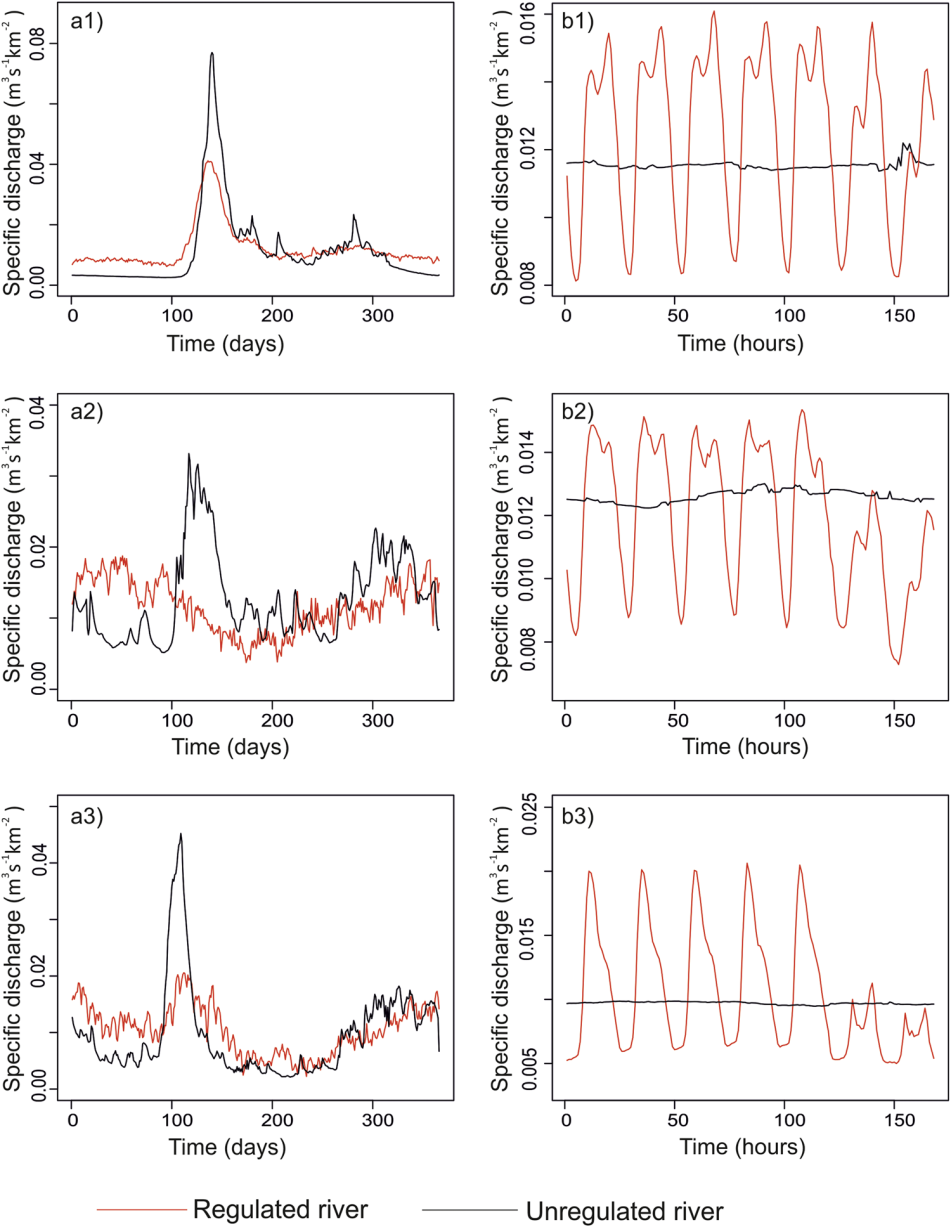
Aggregated mean long term regulation (**a**) and aggregated mean short term regulation (**b**) pattern in terms of specific discharge on large (1) medium (2) and small river (3) and that of comparable unregulated stations. Taivalkoski (regulated), Ounasjoki at kongas (unregulated) on daily scale (**a1**) and hourly scale (**b1**). Montta (regulated), Sanginjoki (unregulated) on daily scale (**a2**) and hourly scale (**b2**). Kyröskoski (regulated), Vakkola (unregulated) on daily scale (**a3**) and hourly scale (**b3**). The x-axis shows (**a1**–**a3**) show days of the year and (**b1**–**b3**) hours of the day.

Variations in specific discharge of regulated rivers were compared against those in rivers with similar natural conditions at daily and subdaily resolution, in order to reveal subdaily fluctuation patterns. There were large deviations between flow patterns of these river systems at daily and subdaily resolution (Fig. 5).

### Effect of catchment and hydropower plant properties on hydropeaking

**Figure 6:**
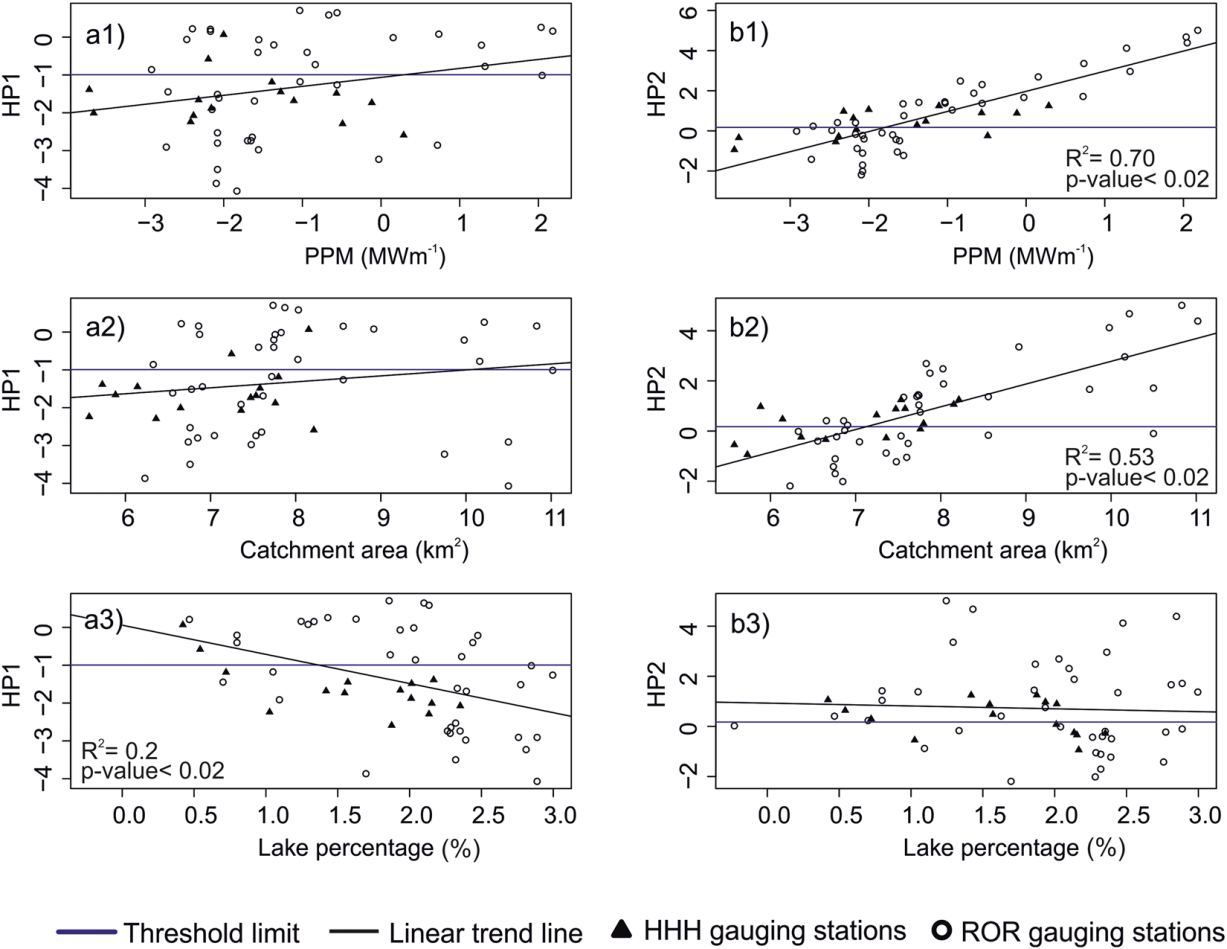
Scatter plot between log transformed values of HP1 (**a**) and HP2 (**b**) with Range of PPM (1), catchment area (2), lake percentage (3) across the studied area. We further divided studied rivers as high-head hydropower plants (HHH), and run-of-the-river hydro power plants (ROR). Circular points show values for ROR type gauging stations and triangular points for HHH type gauging stations. Horizontal line going across the plots are threshold limit values for HP1 and HP2 and black line is the linear trend line. R squared and p-values are shown for variables displaying a statistically significant trend.

Catchment area, installed capacity of the hydropower plant per meter head of the dam (PPM), and lake percentage varied widely for the rivers studied (Fig. 6).

**Figure 7:**
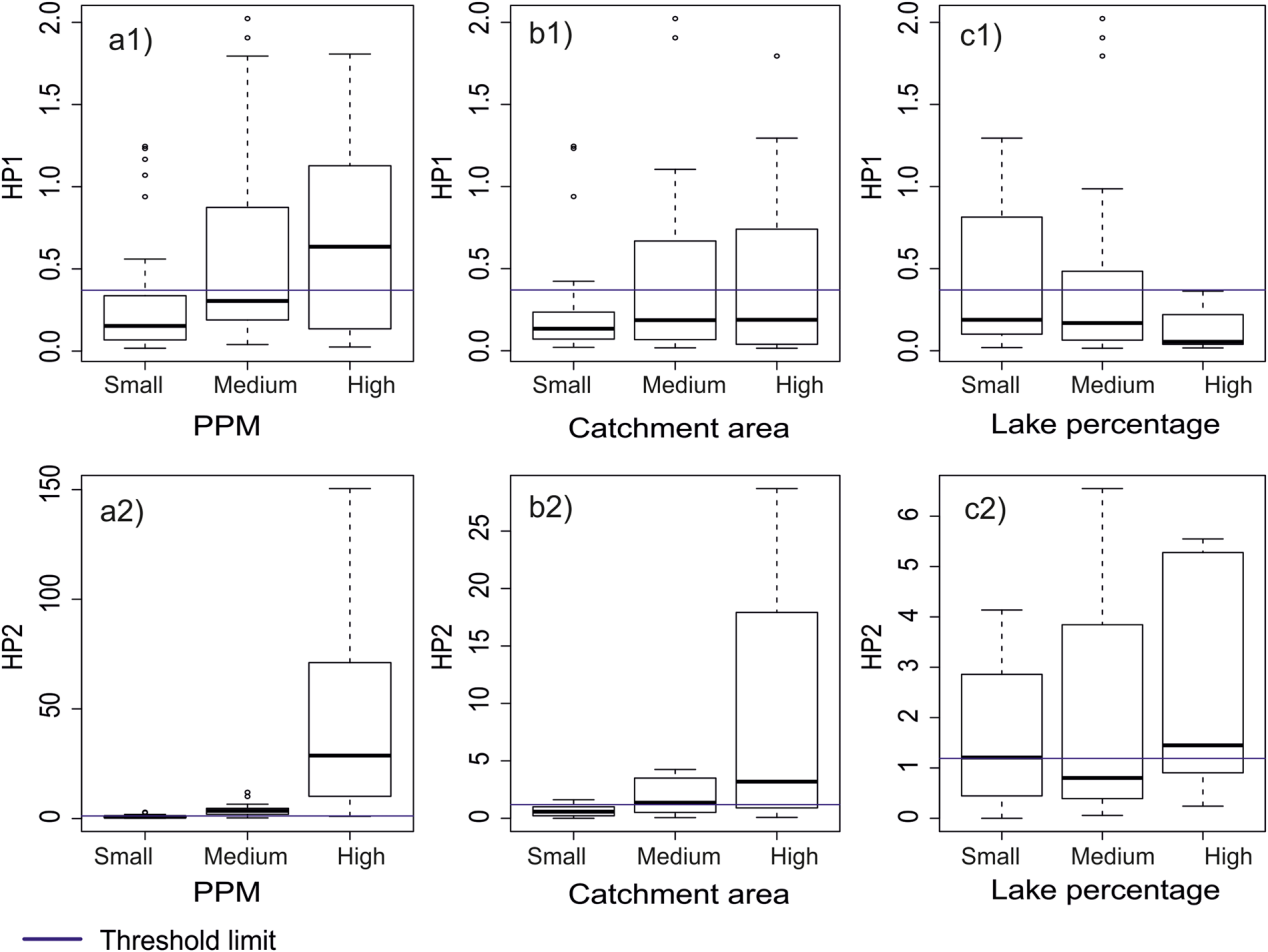
Box-and-whisker plots for the daily averaged values of HP1 (1) and HP2 (2) for small, medium and large PPM (**a**), Catchment area (**b**) and Lake Percentage (**c**). Figure shows the median (dark horizontal line), percentiles 75 and 25 (edges of boxes), percentiles 90 and 10 (end of vertical lines), and outliers (circles). Horizontal line going through all the boxplots are threshold limit values for HP1 and HP2. Note the different sclaes on the y-axis for the HP2 plots.

Both HP1 and HP2 were found to be positively correlated to both the installed capacity of the power plant (PPM) and the catchment area, indicating an induced variability in discharge for more energy producing plants and for larger catchments. For both variables, this correlation was found to be stronger for HP2 compared to HP1 (Fig. 6). Lake percentage displayed contrasting patterns for the two indices where HP1 was found to be inversely proportional to lake percentage while the analyses did not display any correlation for HP2. Moreover, gauging stations at hydropower plants that generated high PPM also showed higher median and interquartile range (IQR) for both HP1 and HP2 (Fig. 7). Larger catchments across the study area also exhibited higher median and IQR for HP1 and HP2. An increase in lake percentage of a catchment increased the median and IQR for HP1, but decreased the median and IQR for HP2 (Fig. 7). Calculated HP1 and HP2 threshold values (shown as horizontal lines in Figs 6 and 7) served a reference to determine the extent of variation in median values of HP1 and HP2.

**Figure 8:**
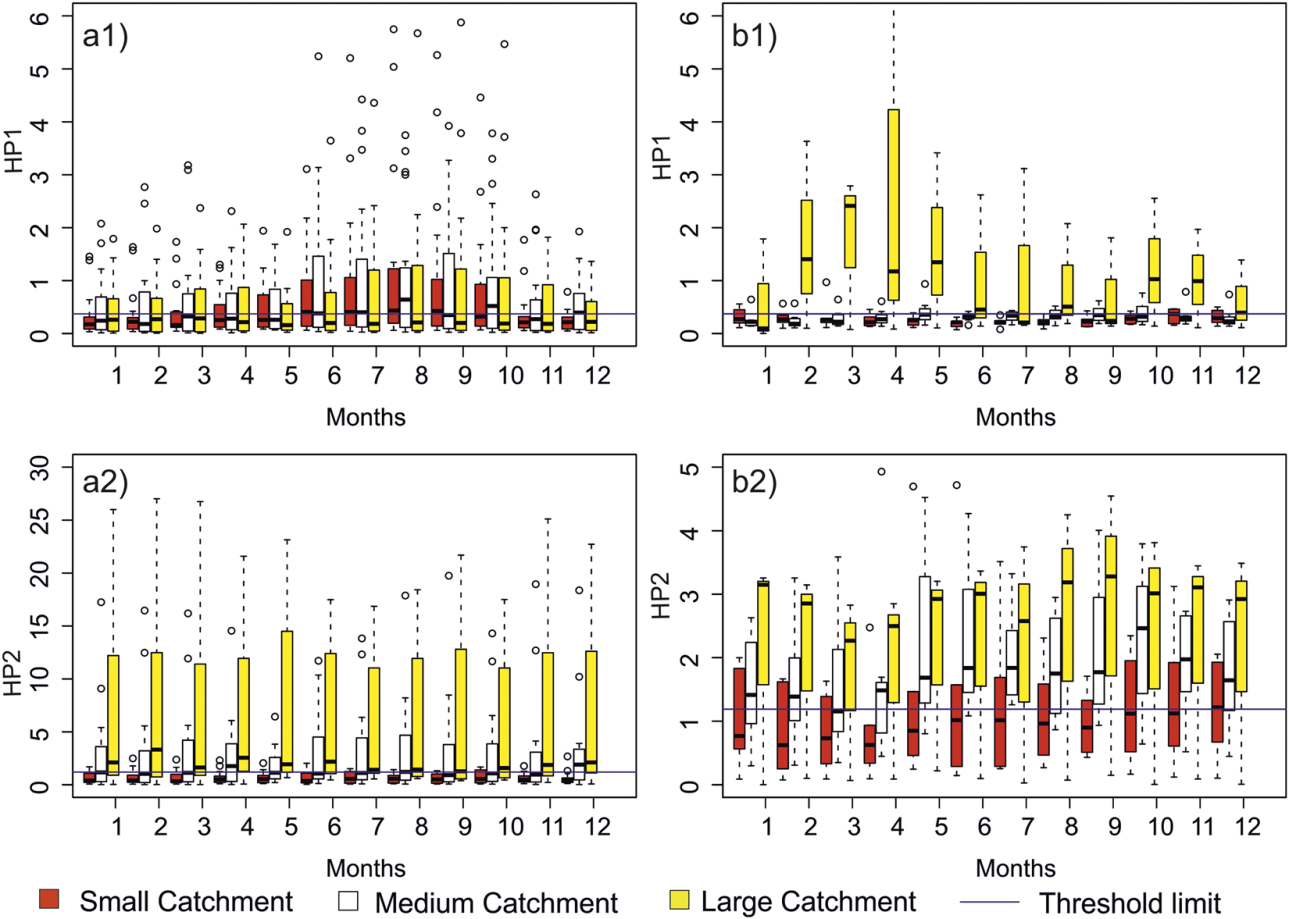
Box-and-whisker plots for the daily averaged values of HP1 for ROR dams (**a1**), HP1 for HHH dams (**b1**), HP2 for ROR dams (**a2**) and HP2 for HHH dams (**b2**) for small, medium and large river catchments. Months on X-axis are in the order of January (1) to December (12). Each month has 3 box-and-whisker plots one for each category of catchment sizes. Figure shows the median (dark horizontal line), percentiles 75 and 25 (edges of boxes), percentiles 90 and 10 (end of vertical lines), and outliers (circles). Horizontal line going through all the boxplots are threshold limit values for HP1 and HP2. Note the different scales on the y-axis for figures (**a2**,**b2**).

**Figure 9:**
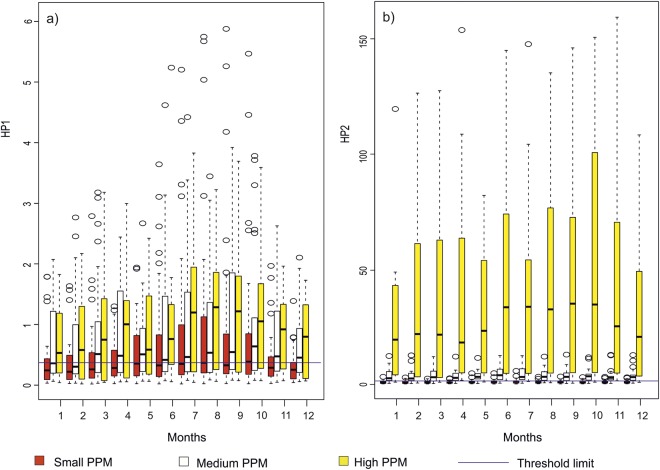
Box-and-whisker plots for the daily averaged values of HP1 (**a**) and HP2 (**b**) for small, medium and large PPM size. Months on X-axis are in the order of January (1) to December (12). Each month has 3 box-and-whisker plots one for each category of PPM sizes. Figure shows the median (dark horizontal line), percentiles 75 and 25 (edges of boxes), percentiles 90 and 10 (end of vertical lines), and outliers (circles). Horizontal line going through all the boxplots are threshold limit values for HP1 and HP2.

Overall, rivers with High-head hydropower plants (HHH) showed lower hydropeaking values than those with Run–off-the-river (ROR) plants (Fig. 8). Seasonal analysis revealed that rivers with ROR plants showed higher hydropeaking values during summer and autumn and lower hydropeaking values during winter and spring (Fig. 8). Rivers with HHH plants had higher hydropeaking values during spring and autumn months and low hydropeaking values in winter and summer months (Fig. 8). Catchment size did not have any effect on HP1 values for rivers with ROR hydropower plants, but rivers with HHH plants showed considerably higher hydropeaking for catchments greater than 3000 km^2^ area (in total 28 catchments; Fig. 8). Rivers with ROR plants had higher HP2 values than rivers with HHH plants in summer and winter months, but slightly lower values during spring and autumn months. Unlike HP1, for HP2 an increase in catchment size resulted in much higher values. Rivers with HHH hydropower plants had comparatively smaller values for HP2, with no relationship to catchment size. The level of PPM did not affect HP1, but rivers with higher PPM had considerably higher HP2 values in all months (Fig. 9).

### Changes in hydropeaking in recent years

**Figure 10:**
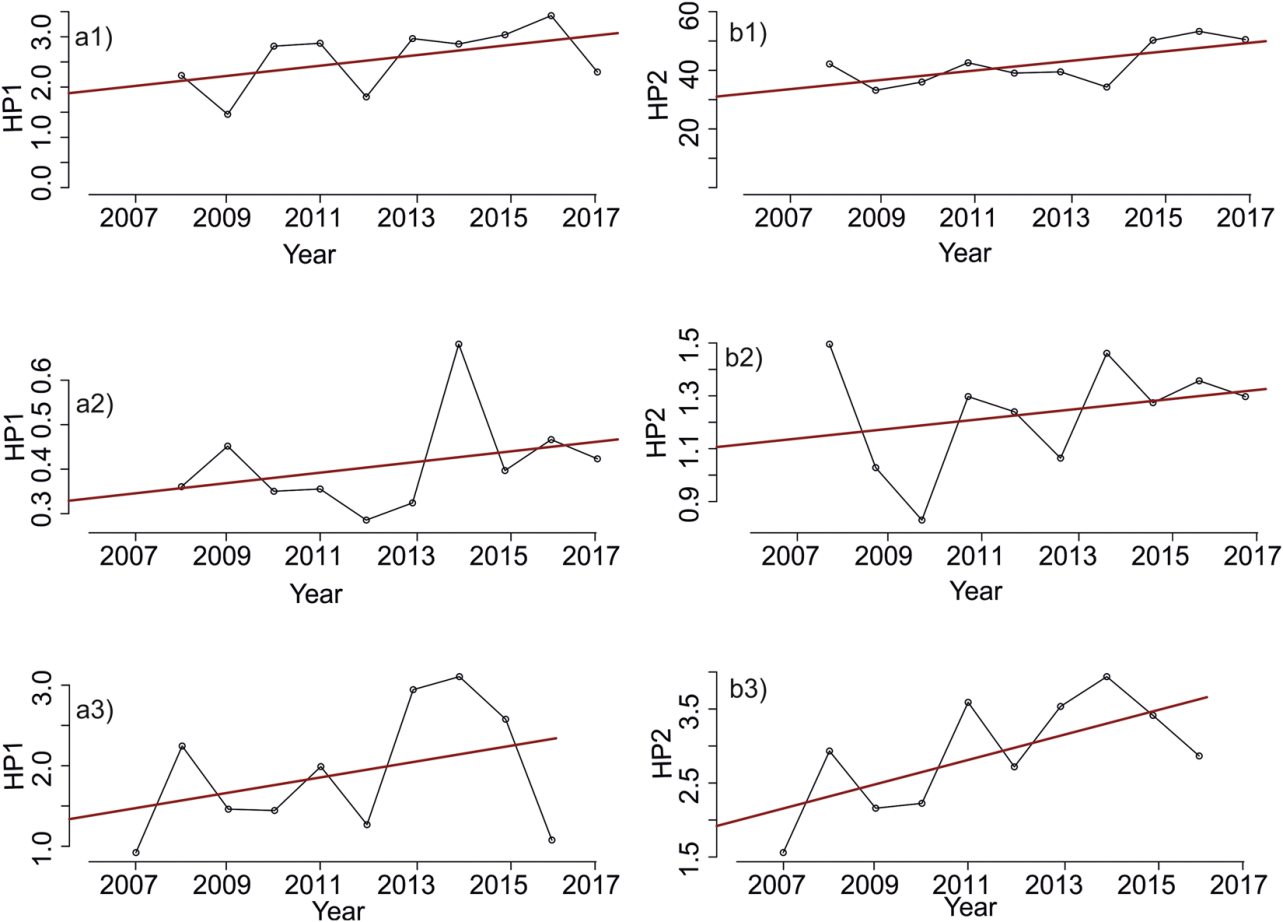
Annual hydropeaking trend of HP1 (**a**) and HP2 (**b**) at Pamilo (1), Vatajankoski (2) and Sokna kraftverk (3).

Of the 92 regulated river gauging stations analyzed, 54 had more than five years of hourly discharge data, and 34 (63%) of those 54 stations showed a significant trend (Mann-Kendall trend test) for HP1, while 40 (74%) showed a significant trend for HP2. Around 50% of the statistically significant gauging stations showed a positive trend slope for HP1, with Sen’s slope value ranging between 0.00007 and 0.087 annually. More than 63% of statistically significant stations showed a positive trend slope for HP2, with annual Sen’s slope value ranging between 0.0003 and 1.2. The remaining stations showed a statistically significant decreasing trend. In the Nordic countries, especially in Finland, there has been a major increase in wind power production since 2013^41^. For all the regulated Finnish rivers studied, there was a statistically significant difference between the periods 2014–2017 and 2011–2013 for both HP1 (Mann-Whitney U-test, p < 0.001) and HP2 (p < 0.001). The sites Pamilo, Vatajankoski (Finland) and Sokna (Norway) showed the highest values for Sen’s slope (Fig. 10).

## Discussion

This study is the first comprehensive evaluation^41^ of hydropeaking in the Nordic countries across different landscapes, catchment types and sizes, and types of hydropower using high-resolution hourly data covering multiple years. According to the results, hydropeaking: (i) Occurs at considerably high levels in the region, (ii) varies with lake percentage (based on HP1’s significant variation with lake percentage), hydropower dam type, and catchment size, and (iii) shows substantial seasonal variation. Hydropeaking indicator HP1 showed higher values for years with low mean flow than the years with high mean flow. This could indicate that hydropeaking is more intense at regulated sites during dry years, when there may already be challenges in maintaining ecologically critical environmental flows. Rivers with larger catchment size and run-of-river (ROR)-type hydropower plants showed higher values of HP1 and of ramping rate indicator HP2 than smaller catchments. Because of the lack of data from highly regulated sites in Sweden, hydropeaking values for Swedish rivers were low. However, there is a high probability that other rivers in Sweden are more affected by hydropeaking, as shown in earlier conducted studies^21,40^.

The capacity of the Nordic power system to store energy in larger reservoirs has a cushioning effect on prices^42^. It is used to secure Nordic energy market demand for flexible energy, which results in higher HP2. This indicates that larger catchments are used to a large degree to balance demand, which causes hydropeaking and unstable flow conditions in the river systems. However, smaller river systems are also sensitive to hydropeaking and our analysis indicated significant difference between HP1 and threshold values in river systems having catchment area of less than 500 m^2^. Many high head hydropower (HHH)-type plants in Nordic river systems still operate on a base load, and hence seasonal analysis is important for their bypass reaches. However, Nordic river systems rarely suffer from severe droughts and thus evaluations of environmental flow conditions, especially at ROR plants, should focus on hydropeaking and not on analyzing daily or monthly flow conditions. While hydropower is highly important for energy markets and in providing a flexible energy source, hydropeaking should be further examined and discussed in a Nordic perspective.

We found considerable variation in the seasonal distribution of hydropeaking, with higher values in summer and autumn months when power production and consumption are at their lowest in the Nordic countries. Winter months showed a lower degree of hydropeaking, which could be due to consistently higher energy demand requiring more even production over the day and thereby more stable river flow. Thus, for the long term regulation at the heavily regulated stations in Finland there is a clear increase in the winter discharge^38^, which can be seen from Fig. 5(a1–a3) where specific discharge for winter Julian days is higher in regulated rivers than unregulated rivers. Low levels of hydropeaking during high energy production season could mean that during these periods the power plants rarely shut down their turbines to meet the constant high energy demand. Low HP2 and high HP1 during March and April could mean a continuous change in sub-daily flow discharge at a considerably small rate of change. Flow variations during this time of the year follow a more natural seasonal flow pattern, because spillways of dams are opened to release large amounts of flood water which cannot be contained or channeled for electricity production. The HP1 and HP2 values for ROR-type power plants indicated higher hydropeaking during low flow months (summer and autumn). This might be the result of higher demand for adjustment energy in markets or due to smaller available water (low flow) in rivers causing more sensitive conditions for hydropeaking. It could also be due to regulations to maintain stable ice cover in river systems in order to prevent formation of fragile ice. Predictability and seasonality are both important for river ecology^32,43^ and for recreational needs. Continuous sub-daily discharge variation that is contrary to the natural regime may in some cases cause loss of habitat availability and degraded ecological conditions

Ramping rates were higher for ROR-type plants than HHH-type plants throughout the year, without any considerable seasonal differences. This could be because the small head of ROR plants requires a higher discharge of water to generate the same amount of power as obtained from a HHH plant. Moreover, in most HHH only a part of the total flow is regulated, thus causing dampening of hydropeaking. There was no significant effect of catchment size and power generated per meter (PPM) on HP1, but HP2 was significantly higher for larger catchment areas and higher PPM. This may indicate a stronger relationship between hydropower plant size and ramping rate than between plant size and net intraday discharge difference.

Over the past decade, wind power production has risen in northern Europe. In 2014, wind power accounted for about 39% of total energy in Denmark^44^ and 8% in Sweden^45^. Wind power constituted less than 2% of the total power production in Finland and Norway until 2016^40,46^. In light of the EU goal of achieving 20% of energy from renewables (2009/28/EC), there is clear desire and goal in the Nordic countries to increase the percentage in future^47^. Thus renewable energy production has now increased in the Nordic countries, especially production of wind power, e.g., in Finland from 259 MW in 2012 to 1533 MW in 2016^40^. The Nordic countries share ‘Nord pool’, an integrated energy market. Due to the increased shares of variable renewable energy, there could be an increase in the use of hydropower as a primary and load balancing source for future energy markets. In the present analysis, we observed that hydropeaking is already at high levels in many Nordic rivers and that there is an increasing trend in HP1 for 63% of rivers with >5 years of observations and in HP2 for 74% of these rivers. This indicates that river regimes have been experiencing increasing levels of hydropeaking in recent years. Furthermore, in Finnish rivers the mean and variation in hydropeaking values have significantly increased since 2013. Thus hydropeaking has increased in recent years, possibly due to increased production of renewable energy and the need for a load balancing source. This indicates a clear need for optimization of hydropower operations in the Nordic countries, as hydropower will continue to play an increasingly significant role in future energy markets. Different scenarios for carbon-neutral power systems in the Nordic countries show an enhanced role of Norwegian hydropower as a load balancing source^48,49^. It is also worth mentioning that future energy export plans (mainly by Norway) mainly is concerned with doing peaking between reservoirs, which would alleviate the current levels of hydropeaking. Therefore, the impacts of using hydropower as a load balancing source of energy clearly need to be investigated more thoroughly, to help policy makers set limits for environmentally sustainable choices. Hydropeaking can have different effects depending on downstream channel morphology and the receiving water body. For example, the effect of hydropeaking occurring downstream from a hydropower dam outlet close to a lake or the sea may differ from that of hydropeaking in headwaters. Our analysis did not consider local aspects, but in management of hydropeaking these issues need to be taken account. It is possible that higher hydropeaking can be permitted in river sections where it does not have major impacts on downstream conditions, or in hydropower plants with outlets in lakes or in the ocean.

## Conclusions

Driving forces for hydropeaking in Nordic rivers were examined using extensive datasets from 157 sites with high-resolution hourly data. The results showed that hydropeaking occurs at considerably high levels on these rivers, with an increase in hydropeaking over the past decade and especially in the past few years. The results also revealed an ongoing upward trend in hydropeaking levels on many regulated rivers in the study region. The recent significant increase in wind power may require an increase in hydropower as a balancing source, which could further increase hydropeaking. Run-of-river (ROR) hydropower plants constructed on rivers with small elevation head were found to have higher hydropeaking than high head hydropower (HHH) plants. Sub-daily flow variation and ramping rate displayed a positive correlation with power per meter available head. Hydropower operations in the Nordic region thus need to be optimized using an integrated economic and ecological framework, in order to address the problem of high levels of hydropeaking in regulated river systems.

## Material and Methods

### Study area and data source

Discharge data from 157 (80 regulated and 77 unregulated) gauging stations or hydropower dams on major pristine and regulated rivers spread across Norway, Sweden, and Finland were collected and analyzed (Fig. 1). Discharge data at hourly resolution (m^3^ s^−1^) were acquired from the Finnish Environmental Institute (SYKE), Swedish Meteorological and Hydrological Institute (SMHI), and The Norwegian Water Resources and Energy Directorate (NVE). Time span of flow rate varied from one year to 10 years for regulated stations and to 20 years for unregulated gauging stations (up to the end of 2017). Hourly discharge data from the SYKE database were unchecked and hence needed correction. This was done using the following equations.

1 $${{\rm{Q}}}_{{\rm{diff}}}={{\rm{Q}}}_{{\rm{daily}}{\rm{unchecked}}}-{{\rm{Q}}}_{{\rm{dailychecked}}}$$

2 $${{\rm{Q}}}_{{\rm{hourly}}{\rm{corrected}}}={{\rm{Q}}}_{{\rm{hourly}}{\rm{unchecked}}}-{{\rm{Q}}}_{{\rm{diff}}}$$

where Q_daily unchecked_ is the daily aggregated mean discharge calculated from unchecked hourly discharge data, Q_daily checked_ is the checked daily discharge value available from the SYKE database, Q_diff_ is the difference between checked and unchecked daily discharge data, and Q_hourly corrected_ is the corrected hourly discharge value.

The flow data were clustered based on catchment size (which varied between 200 and 50 000 km^2^), lake percentage (which varied between 1% and 20%), and power generated per meter (PPM), defined as installed capacity of the hydropower plant per meter head of the dam (which varied between 0.02 and 0.6 MWm^−1^). For more detailed analysis, we divided these properties into three categories, small, medium, and large, based on: Catchment area 0–1000, 1000–3000, and >3000 km^2^ respectively, lake percentage 0–5, 5–10 and >10% respectively, and PPM 0–0.2, 0.2–1, and >1 MW/m^−1^ respectively. Catchment area (km^2^) for each measurement station was calculated using upstream catchment boundaries determined from digital elevation model (DEM, 25 × 25 m resolution; European Environment Agency). Lake surface area (km^2^) and percentage in catchment (%) were calculated from Corine database (European Environment Agency).

To analyze both long- and short-term regulation patterns in different sizes of rivers, specific discharge (discharge per unit catchment area) for these rivers was compared. For this, the following regulated stations were arbitrarily chosen: Taivalkoski (large), Montta (medium), and Kyröskoski (small). Specific discharge flow regime at these regulated stations was compared with that at a comparable natural reference station based on similar location and climate. Vakkola, Ounasjoki at köngäs and Sanginjoki, respectively, were chosen as the natural reference stations. Hydropower plant types present on the studied rivers were broadly categorized into two types; (1) Run-of-river (ROR) (constructed in the river with the hydropower plant installed in or close to the dam), and (2) high-head hydropower (HHH) (dam and hydropower plant with high elevation connected by tunnel). In total, 19 stations had HHH-type and around 61 ROR-type hydropower plants.

## Methods

### Hydropeaking indicators

To study hydropeaking properties in catchments, we used the indices developed by Carolli *et al*. in 2015^34^. The first indicator, HP1, is a dimensionless measure of the magnitude of hydropeaking and is the annual median of daily *HP1*_*i*_ values, calculated as the difference between maximum and minimum discharge over the *i*th day, normalized by the mean daily discharge. It is expressed as:

3 $$HP{1}_{i}=\frac{{Q}_{max,i}-{Q}_{min,i}}{{Q}_{mean,i}},\,\,i\in [1,365]$$

4 $$HP1=median(HP{1}_{i})$$

5 $${\rm{HP}}{1}_{{\rm{monthly}}}={\rm{aggregated}}\,{\rm{monthly}}\,{\rm{mean}}({\rm{HP}}{1}_{i})$$

where subscript _i_ is day of the year, Q_max,i_ and Q_min,I_ are the maximum and minimum discharge, respectively, and Q_mean,i_ is mean daily discharge.

The second indicator, HP2, measures ramping rate, i.e., the temporal rate of discharge changes^34^, and is defined as:

6 $${(HP{2}_{k})}_{i}=(\frac{{\rm{\Delta }}{Q}_{k}}{{\rm{\Delta }}{t}_{k}})={(\frac{{Q}_{k}-{Q}_{k-1}}{{t}_{k}-{t}_{k-1}})}_{i},\,i\in [1,365]$$

7 $$HP{2}_{i}={P}_{90}|{(HP{2}_{k})}_{i}|;$$

8 $$HP2=median(HP{2}_{i}).$$

9 $${\rm{HP}}{2}_{{\rm{monthly}}}={\rm{aggregated}}\,{\rm{monthly}}\,{\rm{mean}}\,({\rm{HP}}{2}_{{\rm{i}}})$$

where subscript _k_ refers to each available discharge datum (e.g., [1 ≤ k ≤ 24] for data sampled hourly) and |…| denotes absolute value. HP2 is computed as the annual median of daily values of HP2_i_, which is the 90th percentile (P_90_) of the discretized time derivative of the instantaneous stream-flow series. HP2 is a dimensional parameter and is expressed in m^3^ s^−1^ h^−1^. The 90^th^ percentile, P_90_, was arbitrarily chosen as a measure of the daily rate of change because it is a conservative estimation of the cutoff value for extreme high flow events and allows exclusion of possible error measurements. By using the absolute value of P_90_, ramping rates of the hydrographs in both directions, i.e., the increasing and falling limb, were taken into account. Finally, annual median values were used to characterize each gauged station with a distinctive yearly value for both indicators.

### Hydropeaking thresholds and hydropeaking pressure classes

For quantification of hydropeaking, a threshold (TR) was set for each indicator: TR_HP1_ and TR_HP2_^34^. These thresholds were calculated based on 70 natural discharge datasets for Swedish, Finnish, and Norwegian rivers, using a non-parametric method^50^ in order to avoid a priori assumptions on normality in data distribution. The values of the two thresholds corresponded to the values of the two estimators which separated the outliers from the rest of the unpeaked distribution.

The chosen threshold values, as calculated by Carolli *et al*.^34^ were defined as:

10 $$T{R}_{HP1}={P}_{75}(HP{1}_{i}^{unp})+1.5({P}_{75}-{P}_{25})(HP{1}_{i}^{unp}),$$

11 $$T{R}_{HP2}={P}_{75}(HP{2}_{i}^{unp})+1.5({P}_{75}-{P}_{25})(HP{2}_{i}^{unp}),$$

where $HP{1}_{i}^{unp}$ and $HP{2}_{i}^{unp}$ are the daily values of the two indicators for unpeaked stream gauges and P_75_ and P_25_ are the 75^th^ and 25^th^ percentile of the distribution, respectively.

After the identification of thresholds, values of HP1 and HP2 were classified according to the following four levels^34^.

1. Class 1: Low level of hydropeaking. HP1 < TR_HP1_ and HP2 < TR_HP2_.
2. Class 2a: Moderate level of hydropeaking. HP1 > TR_HP1_ and HP2 < TR_HP2_.
3. Class 2b: Moderate level of hydropeaking. HP2 > TR_HP2_ and HP1 < TR_HP1_.
4. Class 3: High level of hydropeaking. HP1 > TR_HP1_ and HP2 > TR_HP2_.

### Trend analysis

The Mann-Kendall trend test was used to quantify the significance of trends in HP1 and HP2 over time, and Sen’s slope estimator was used for estimating the slope of the trends. We identified the year 2013 as the point when wind power production in the Finland rose sharply^40^. The Mann-Whitney U test was used to assess the significance of shifts in the median of HP1 and HP2 time series pre- and post- 2013.

## Data Availability

The datasets generated during and/or analysed during the current study are available from the corresponding author on reasonable request.
